# Epitope specificity appears to be an important determinant of in vivo killing ability of simian immunodeficiency virus (SIV)-specific CD8+ T Cells

**DOI:** 10.1186/1742-4690-9-S2-O6

**Published:** 2012-09-13

**Authors:** L Pozzi, A Carville, V Varner, J Sen, D Knipe, A Kaur

**Affiliations:** 1 Harvard Medical School, Southborough, MA, USA

## Background

CD8+ cytotoxic T lymphocytes (CTL) are a critical component of antiviral immunity and play an important role in the control of lentiviral infection.

## Methods

We have developed an in vivo CTL assay to directly measure the killing capacity of MHC-restricted SIV-specific CTL in rhesus macaques (RM). In order to evaluate the in vivo efficacy of different epitope-specific CTL, we compared the in vivo killing capacity of Mamu-A*02-restricted Nef YY9 and Gag GY9 CD8+ T lymphocytes in three RM vaccinated with a recombinant HSV prime/ DNA boost SIV vaccine regimen.

## Results

Tetramer frequencies of Mamu-A*02-restricted Nef YY9-specific CD8+ T-cells were at least 10-fold higher than Mamu-A*02-restricted Gag GY9-specific CD8+ T-cells in individual animals both pre- and post-challenge. Prior to SIV challenge, both CTL populations showed poor and incomplete killing (22-35%) of target cells over 18 hours. Seven weeks post-challenge, there was a marked increase in the CTL killing capacity of both Nef YY9 and Gag GY9-specific CTL with 26-38% killing occurring over the first two hours and up to 100% killing over 18 hours. Surprisingly, Gag GY9-specific CTL consistently showed equivalent or greater in vivo killing when compared to Nef YY9-specific CTL even though the percentages of IFN-γ secreting and degranulating cells upon peptide stimulation were comparable.

## Conclusion

These data suggest that epitope specificity rather than tetramer frequency determines the ability of SIV-specific CTL to kill infected cells. Taken together, these data may have important implications for the development of a successful HIV vaccine.

